# Attentional Modulation of Brain Responses to Primary Appetitive and Aversive Stimuli

**DOI:** 10.1371/journal.pone.0130880

**Published:** 2015-07-09

**Authors:** Brent A. Field, Cara L. Buck, Samuel M. McClure, Leigh E. Nystrom, Daniel Kahneman, Jonathan D. Cohen

**Affiliations:** 1 Princeton Neuroscience Institute, Princeton University, Princeton, New Jersey, 08540, United States of America; 2 Department of Psychology, University of California, San Diego, La Jolla, California, 92093, United States of America; 3 Department of Psychology, Stanford University, Stanford, California, 94305, United States of America; 4 Woodrow Wilson School, Princeton University, Princeton, New Jersey, 08540, United States of America; 5 Department of Psychology, Princeton University, Princeton, New Jersey, 08540, United States of America; 6 Department of Psychiatry, University of Pittsburgh, Pittsburgh, Pennsylvania, 15213, United States of America; Duke University, UNITED STATES

## Abstract

Studies of subjective well-being have conventionally relied upon self-report, which directs subjects’ attention to their emotional experiences. This method presumes that attention itself does not influence emotional processes, which could bias sampling. We tested whether attention influences experienced utility (the moment-by-moment experience of pleasure) by using functional magnetic resonance imaging (fMRI) to measure the activity of brain systems thought to represent hedonic value while manipulating attentional load. Subjects received appetitive or aversive solutions orally while alternatively executing a low or high attentional load task. Brain regions associated with hedonic processing, including the ventral striatum, showed a response to both juice and quinine. This response decreased during the high-load task relative to the low-load task. Thus, attentional allocation may influence experienced utility by modulating (either directly or indirectly) the activity of brain mechanisms thought to represent hedonic value.

## Introduction

Intuitively, attention seems to alter our subjective valuation of pleasurable and unpleasant experiences. Consider driving a sports car. When thinking about how happy driving it makes you, it is likely you would experience pleasure. This sense of pleasure is seemingly missing when driving to work absentmindedly or engaging a passenger in conversation. This effect of attention on valuation may be important for a variety of domains, including studies of well-being. Such studies often rely on self-report as an index of current state. However, if self-report draws attention to valuative processes, this could impact their function and thus introduce a bias in sampling.

While introspection suggests that attention can modulate our experienced utility, testing for such effects is an experimental challenge. The standard method of self-report is problematic since, as suggested above, directed attention is required to answer the question. Reporting pleasure as moment-utility may help mitigate this problem somewhat, since responding may become more automatic (that is, less reliant on attention). However, it is unclear the extent to which such reports are automatic, which at best introduces additional variance into measurements. To avoid these problems, we take advantage of findings indicating that reward value may be measured directly from ongoing brain activity using functional magnetic resonance imaging [fMRI; 1].

The ventral striatum has been implicated in representing hedonic value [1–6]. This structure responds to primary (*i*.*e*., unconditional) reward and punishment at first lifetime exposure [7]. Its activity correlates with subjective ratings of liking [8] as well as orofacial muscle contractions associated with pleasure [9]. There remain questions as to whether it reflects hedonic value primarily in a valenced way (i.e., positive vs. negative value), or in an unvalenced way that is sometimes referred to as salience [10–15].

Whether ventral striatal activity reflects the sign and/or salience of hedonic stimuli, we assumed that either could be used to index the magnitude of response to hedonic stimuli, and predicted that attentional load would reduce the magnitude of these responses as measured using fMRI. To test this, we combined hedonic stimulation using primary appetitive and aversive stimuli with a task that could be used to manipulate attentional load. Allocation of attention is generally believed to rely on limited capacity mechanisms responsible for working memory and cognitive control [16–21]. Thus, to manipulate attentional load, we used the *n*-back task [22,23] that has been used widely to manipulate the engagement of working memory [22–26]. Subjects alternately performed either a low-load (0-back) or high-load (3-back) version of the *n*-back task [24] while their brains were scanned using fMRI. As the subjects engaged in these tasks, controlled aliquots of either fruit juice (appetitive) or quinine hydrochloride (HCl) solution (aversive) were orally delivered during the inter-trial-interval.

## Methods

### Participants

This study was approved by Princeton University’s Institutional Review Panel prior to commencement. We obtained informed consent in writing from seventeen right-handed, healthy subjects (fourteen female, three male; mean age 21.9; age range 18–27). All subjects practiced the behavioral task prior to scanning. To control for thirst we requested that subjects drink nothing for three hours prior to the scan. They ate five Nabisco Premium Saltines crackers approximately ten minutes prior to scanning.

### Cognitive Task

To manipulate attention, we engaged subjects in an attentionally-demanding working memory task [16–21]. For high and low attentional load, we used the 3-back and 0-back versions of the *n*-back task [22–26], respectively. Subjects viewed a pseudorandom sequence of letters, one letter appearing every 18 s and lasting for 500 ms. Half of the letters were targets. In the 0-back task, a target letter appeared at the beginning of the run. Subjects then responded by indicating whether each letter presented in the sequence matched that original letter. In the 3-back task, the target was the letter that was presented three letters earlier in the sequence. Thus, the target letter was continually changing. Of the nontargets, half were foils. These were repeats of letters that occurred between 0 and 4 back, but never 0 back in the 0-back task and never 3 back in the 3-back task.

For the first eleven subjects, responses were recorded only for the first 1.25 s post stimulus onset. However, we suspected that subjects were occasionally taking longer than 1.25 s to respond in the high-load task. To assure that subjects were maintaining high response accuracy, we extended the response recording period to 3.0 s for the remaining 6 subjects. This modification had no impact on subjects’ performance (see S1 Text).

### Hedonic Stimuli

Two nine-yard sections of vinyl tubing (1/4” outer diameter, 1/8” inner diameter) were fitted through a pacifier shield such that one end of each tube was flush with the tip of the pacifier bulb. When placing the pacifier in the mouth, both tubes rested near the tongue. The other ends of the tubes were attached to needleless syringes, one filled with juice and the other quinine solution. The syringes were compressed by pumps (Harvard Apparatus), which were controlled by a computer running E-Prime software (Psychology Software Tool, Inc.). The latency of injection due to line compliance was about 0.75 s.

During each trial, 0.5 ml (1 ml/s for 0.5 s) of either juice (Hi-C Boppin’ Strawberry) or quinine solution (0.10 mM quinine dihydrate HCl; Sigma-Aldrich) was squirted into the subject’s mouth. This concentration of quinine was substantially below that used in previous imaging studies [27,28]. Pilot testing indicated that the hedonic intensity at 0.10 mM quinine was comparable in magnitude to (but opposite) that of the juice. 30 ml of each were infused over the course of the entire experiment. Subjects were instructed to swallow whenever they detected juice or quinine solution in their mouth.

**Fig 1:**
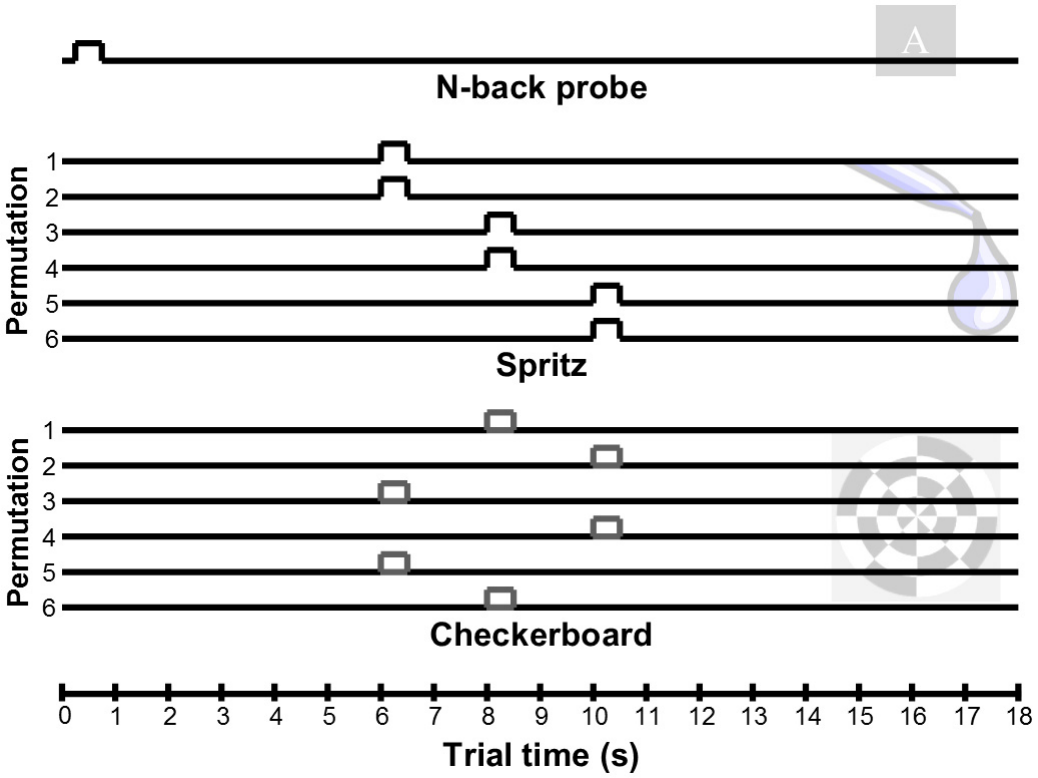
The trial components’ timing. Each trial lasted 18 s. The *n*-back stimulus was presented at 250 ms and subjects were asked to respond immediately. At 6, 8, or 10 s following stimulus onset, subjects received 0.5 ml boluses of either juice or quinine solution in their mouths. On the half the trials (50 percent probability represented by grey line), a flickering checkerboard was shown for 500 ms at 6, 8 or 10 s following stimulus onset, but never when juice or quinine was delivered. The timing of the solution administration and checkerboard were determined by a permutation table (S1 Table), each of the six outcomes of which are displayed here.

There were five scanner runs. Each run involved a block of twelve low-load trials and a block of twelve high-load trials. The ordering of the blocks within each run was pseudorandomly counterbalanced. Fig 1 summarizes the trial design. Each trial was 18 s long. This helped separate the hemodynamic response to the n-back stimulus and the subject’s response to it, which occurred at the beginning of the trial, from the hemodynamic response to the time-randomized delivery of juice and quinine that occurred near the middle of the trial (between 6 and 10 s after onset of the n-back stimulus; see below). The n-back stimulus appeared 250 ms after trial onset and lasted 500 ms. This was followed after several seconds by either juice or quinine delivery and, on half of all trials, also a visual stimulus (8 Hz flickering checkerboard for 500 ms). The visual stimulus was included to assess any non-specific effects of the attentional manipulation (e.g., generalized effects on hemodynamic responses throughout the brain), that might confound the interpretation of any observed effects on hedonic responses. Each of the three event types (juice, quinine, or checkerboard) occurred on half of all trials, at one of three times points (with 1-in-6 odds on a given trial): 6, 8, or 10 s into the trial. The timing of these stimuli was permuted pseudorandomly, such that juice and quinine were never delivered on the same trial, and the visual stimulus was never delivered at the same time as juice or quinine delivery (see S1 Table for further elaboration). There were six juice squirts, six quinine squirts, and six checkerboards during every block.

Afterwards, subjects rated how much they liked the juice and quinine, and how thirsty they were before and after the scan, on an 11-point Likert scale that ranged from -5 to 5 (see S2 Text for the full exit questionnaire).

### FMRI Data Aquisition

MRI scans were acquired with a Siemens Allegra 3.0-T head-dedicated scanner. Anatomical images were acquired using a T1-weighted MP-RAGE protocol. Functional images were acquired using a T2*-weighted EPI (TR = 2000 ms, TE = 30 ms, 64 x 64 matrix, FOV = 192 mm, 3 mm axial slices with 1 mm gap, flip angle = 90°). Five functional runs (223 scans each, including a block each of low and high-load tasks) were conducted for each subject.

### FMRI Preprocessing

Data were analyzed with AFNI [29] software. Standard preprocessing steps were employed. Functional images were slice-time corrected and motion corrected by alignment to the fifth scan of the first run. At this point, one subject was excluded from any further imaging analysis because of excessive head movement (greater than 5 voxels of displacement, while other subjects moved less than 2 voxels). Functional images were corrected for time-series outliers with the AFNI program 3dToutcount, then blurred with a 6 mm FWHM full-width-at-half-maximum Gaussian filter, and spatially transformed to match AFNI’s Talairach-aligned version of the ICBM 452 T1 atlas.

### FMRI Analysis

We performed three different types of fMRI analyses: an event-related analysis that evaluated the relationship between attentional load and BOLD responses to juice and quinine, a region-of-interest analysis that ensured that load-related changes in BOLD responses were regionally specific, and an analysis of the task blocks that confirmed that our paradigm produced results comparable to previous n-back studies. The region of interest analysis involved a functional selection process. We used a Leave-One-Subject-Out procedure so that the functional selection process was statistically independent of subsequent analyses. We discuss these methods in more detail below.

Our primary statistical methods involved event-related fMRI analysis assuming eight event classes of interest: four onset times (*n*-back probe, juice, quinine, and checkerboard stimuli) under each of the two load conditions (low versus high). We created idealized regressors for each of these event types by convolving event onset times with a gamma variate kernel derived using the AFNI GAM default parameters. Within each subject, a general linear model was used to estimate the relative contribution of these regressors to the observed BOLD signal within each voxel (i.e., beta parameter estimates). Nuisance regressors were also included to model known sources of noise, including regressors representing each of the 6 parameters for rigid-body transformations obtained from the motion correction procedure, plus regressors representing a third-order polynomial fit to each continuous block of trials (to remove low frequency drifts).

We then performed group analyses using the eight (non-nuisance) beta parameter estimate maps from each subject. We did this using an ANOVA run within a leave-one-subject-out (LOSO) k-fold cross-validation procedure to select voxels for subsequent contrast analyses. The LOSO procedure ensured that the contrast analyses performed on voxels were statistically independent from the ANOVA voxel selection process itself [30,31]. Specifically, we divided the experimental dataset into 16 different subsamples, with each subsample excluding one subject. That is, for each “fold” of the analysis, the data for a given subject was paired with a subsample that comprised the data from all of the other subjects. In each fold, we performed a 2 X 4 ANOVA to identify regions exhibiting a main effect of load (2 levels: high, low), a main effect of stimulus type (4 levels: juice, quinine, probe, checkerboard), or their interaction; this was a mixed-effects repeated-measures ANOVA with subjects as a random effect. Again, this was performed without including data from the subject left out on that fold. We then submitted to contrast analyses regions that had a voxelwise probability of less than 0.001. Because the ANOVAs in each of the 16 folds produced slightly different results (i.e., statistical maps identifying slightly different sets of activated voxels), we adopted a conservative procedure to produce a single aggregate statistical map across the 16 folds: We computed the strict intersection of the thresholded maps across the 16 folds, retaining only voxels for contrast analyses that appeared in all 16 folds of the LOSO selection process.

We then evaluated the ANOVA main effects and performed contrast tests on voxels identified in the LOSO procedure. Two contrasts were conducted. One tested for voxels sensitive to signed hedonic value and the other tested for the interaction of load with stimulus type. These were used to identify regions comprised of at least thirteen contiguous (nearest neighbor) voxels (540 mm^3^ in Talairach space) that had a voxelwise probability value of less than 0.001. The thirteen contiguous voxel criterion was derived from Monte Carlo simulations using the AFNI program Alphasim. This was used to estimate the family-wise probability of clusters of a given size in a random noise field with the amount of spatial smoothness estimated in our data, and voxel-wise probabilities of p<0.001. This procedure found that clusters of 13 contiguous voxels or more occurred with p<0.05. Thus, we identified such clusters as statistically significant in our analyses.

To visually aid the interpretation of these results, we also estimated each stimulus event’s average impulse response function (i.e. hemodynamic response) using standard Dirac-function-based deconvolution methods [29] over the first 10 s following each event onset. Impulse response functions were averaged across subjects. These hemodynamic response estimates were statistically dependent on the prior use of the contrast for region selection and are thus useful for interpreting the result but not for determining the exact effect sizes [30,31].

To control for whether likely effects of load were global and nonspecific, we also looked for possible modulation of the primary visual cortex's responses to the checkerboard stimuli. We used an AFNI anatomical mask for Brodmann Area 17 (BA17) to identify the correct region. The use of the anatomical mask made for a statistically independent selection process and as such we did not run a LOSO procedure. We ran a group t-test to evaluate the effect of load on the on average across BA17 voxels. Because anatomical masks do not elicit statistical dependence issues, amplitudes for the visual flickering checkerboard for 0- and 3-back blocks can be considered reliable.

In addition to these event-related analyses, we ran a separate analysis comparing task blocks to confirm that the *n*-back task did in fact produce effects on the brain that were comparable with prior reports. The regression model in this case consisted of the motion-correction regressors and a block regressor that coded the 0- and 3-back portion of each run. We identified regions significantly differing in their response to load block, each containing at least thirteen contiguous voxels with a voxelwise probability value of less than 0.001.

### Data and Code Availability

A copy of the data and analysis code can be downloaded from the Princeton University DataSpace service (http://arks.princeton.edu/ark:/88435/dsp0170795988h).

## Results

### Hedonic Ratings

The gustatory stimuli produced expected behavioral effects. The magnitude of hedonic responses was comparable for juice and quinine (S1A Fig). Subjects rated juice likability as 3.24 ± 0.35 (s.e.m.) and the quinine likeability as -3.24 ± 0.35 (s.e.m.). This difference was significant (paired *t*(16) = 9.65, *p* = 3.0 X 10^-08^, difference of means 6.47, 95% confidence interval 5.78 to 7.16). There was also no significant difference in the absolute magnitude of the juice and quinine likability ratings (paired *t*(16) = 0.00, *p* = 1.00), indicating that the magnitude of the likeability ratings were comparable for juice and quinine.

### Replication of Previous n-Back Findings: Block Design Analysis

The *n*-back task also produced expected effects. Slower, less accurate responses confirmed that subjects found the high-load task more challenging (see S1B and S1C Fig for reaction time and accuracy data as well as S1 Text for statistics). We also ran an analysis of the fMRI data using standard methods from the *n*-back literature, contrasting the sustained BOLD levels for blocks of 0- versus 3-back trials (see S2 Fig). These methods and results are comparable to previous studies [23,24], showing increased activity in the 3-back condition in areas commonly associated with attentional demands and working memory load.

### Effect of Load on Event-related BOLD Response

We used a LOSO cross-validation procedure to run an event-related 2 (load) by 4 (stimulus) mixed-effects repeated-measures ANOVA. A number of regions exhibited a main effect of load; that is, had significantly different responses to the stimuli in the 3-back versus the 0-back condition. These included the striatum, insula, parietal cortex, and medial regions of the cortex. These regions are depicted in S3 Fig.

### Effect of Stimulus on Event-related BOLD Response

A number of regions also exhibited a main effect of stimulus (see S4 Fig). We ran a contrast on the areas showing the main effect of stimulus to look for regions sensitive to signed hedonic value (juice/positive versus quinine/negative). To our surprise, there was little evidence of regions that responded differently for positive versus negative values. This included the ventral orbitofrontal cortex, even though it has been associated with appetitive valuation in numerous studies [3,15,32–35]. To address the possibility that this absence of a finding reflected sinus-related signal degradation often found in this region, we conducted an additional region of interest analysis on this area. Using AFNI’s predefined atlas mask for ventral orbitofrontal cortex, we excluded four subjects who had high levels of orbitofrontal signal dropout in the raw EPI images. Analysis of the remaining 12 subjects found trend-level selectivity for juice in comparison to quinine in the left ventral orbitofrontal cortex (see S3 Text).

### Load Versus Stimulus Interaction on Event-Related BOLD Response

**Fig 2:**
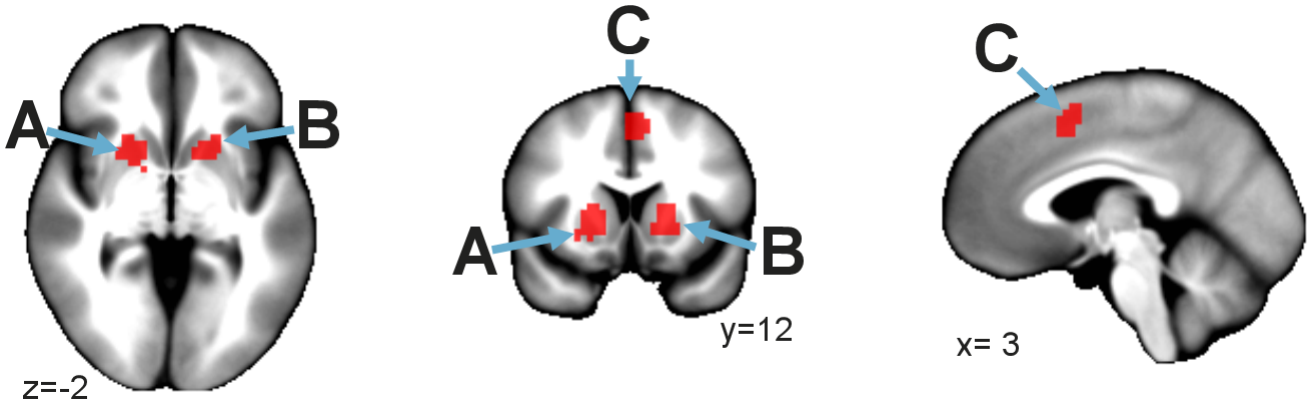
Reward processing regions had a load-related reduction of BOLD response to juice and quinine, but visual stimulation was not modulated. Regions A-C show where there was significant load-related BOLD reduction, and corresponded to the regions listed in Table 1. Region A is in the left striatum. Region B is in the right striatum. Region C is the dorsal anterior cingulate region. Neurological convention.

To test for the influence of the attentional manipulation on stimulus responses, we examined the interaction of load with stimulus. This interaction was significant in regions associated with reward processing (see S5 Fig). Much of this interaction was due to load-related reduction of responses to juice and quinine. Within voxels exhibiting a significant interaction, we contrasted the response to juice and quinine in the 0-back condition versus juice and quinine in the 3-back condition. There were three resulting significant regions: left striatum, right striatum, and a region within the dorsal anterior cingulate (Fig 2 regions A-C). The directionality of the contrast t-statistic indicated that there was a substantial load-related reduction for each region (i.e., 3-back BOLD response was significantly smaller than the 0-back BOLD response). The coordinates for these regions are listed in Table 1. S6A–S6C Fig shows estimated hemodynamic responses for these events, which clearly illustrate that responses were smaller in the three back condition.

**Table 1:** Regions showing load-related reduction of BOLD responses to hedonic stimuli (juice and quinine combined).

		Talairach*					CI^†^	
Region	Volume mm^3^	X	Y	Z	Prob.	Diff. in means	Low	High
Left Striatum	2349	-22.5	7.5	-6.5	3.04E-05	0.00311	0.00203	0.00420
Right Striatum	1782	22.5	16.5	-0.5	7.09E-07	0.00390	0.00231	0.00549
ACC region	1107	1.5	13.5	44.5	5.30E-06	0.00371	0.00261	0.00480

* Center of mass

^†^ 95% confidence interval Bonferroni-corrected for number of ROIs

Finally, we note that the trend-level hedonic responses observed in ventral orbitofrontal cortex were also substantially reduced in the high load relative to the low load condition (see S3 Text for additional discussion).

### Specific Versus Generalized Effects of Attention

**Fig 3:**
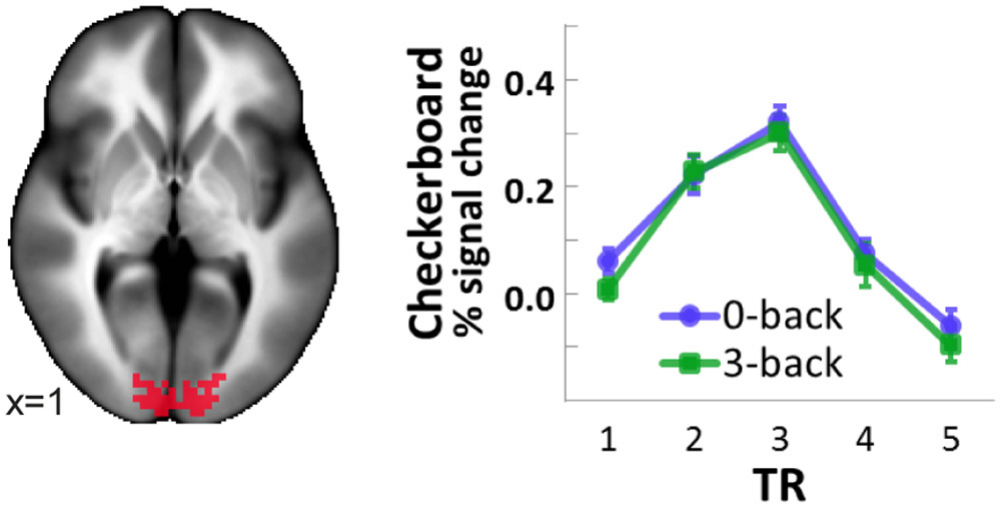
Load did not affect the checkerboard BOLD response. This shows BOLD responses to the flickering checkerboard stimulus within a predefined AFNI anatomical mask for BA 17. This region exhibited no significant *n*-back modulation. Estimated hemodynamic responses are statistically independently of the ROI selection (a predefined anatomical mask). Error bars show s.e.m.

One possible alternative explanation of our findings is that attentional load produced a global, non-specific reduction in responses within the brain—due either to a redistribution of neural activity and/or blood flow responses—that was not specific to the effects of attention on valuation processes. To test this, we evaluated the response in primary visual cortex to a visual checkerboard stimulus. The average checkerboard-driven response of this region was not significantly changed by load (t(15) = 1.137, p = 0.273, difference in means 0.00047, 95% confidence interval -0.00046 to 0.00048). Fig 3 shows this region along with responses in both the low and high-load checkerboard conditions. As a control, we also confirmed that the regions in the ANOVA interaction term showed no checkerboard-load interaction. We ran a contrast of high versus low load on checkerboard-driven responses, but no voxels in the interaction term showed a significant effect of load.

## Discussion

The results of this study demonstrate that a manipulation of attentional load can modulate neural responses to both appetitive and aversive hedonic stimuli, consistent with the introspective experience that attention can influence subjective valuation. This effect was observed most strongly in the striatum. Decades of evidence tie the striatum to hedonic function [1–9]. However, to our surprise, we did not find any robust signed or value-selective activity (that is, areas that responded selectively or oppositely to positively and negatively valenced stimuli). Before considering the implications of this, we should point out that our gradient-echo pulse sequence is susceptible to sinus-related signal degradation in the orbitofrontal cortex. This region has been linked to signed value [11,36–38]. We do not rule out that link. Indeed, the left orbitofrontal cortex did show trend-level value-related responses. However, even there, we observed modulation by attention.

### Value Versus Salience

Our failure to find many regions that differentiated signed value is consistent with the literature, in which areas once thought to represent primarily positive value are increasingly being observed to be responsive to negative value as well. This suggests that responsivity to hedonic salience may be stronger and/or more general than responses to signed value. Such salience may reflect arousal [10,39] or motor planning [40]. Attempts to further validate this would benefit from parameterizing responses to a range of hedonic values including hedonically neutral solutions.

This issue is of particular relevance to the striatum, known to be an important structure in processing appetitive and aversive stimuli. Our findings are in line with other recent fMRI-based reports that have also found evidence of salience responses [10–15]. While much attention focuses on striatal responses to appetitive stimuli, striatal responses to aversive stimuli [11,41–43], including quinine [44], have also been previously demonstrated. Other studies, using direct neuronal recordings, have found that striatal neurons that are selectively responsive to appetitive stimuli are found within a short distance of those selectively responsive to aversive stimuli [45,46]. Thus, a question for future research is whether the pattern of striatal responses observed in imaging studies reflects a genuine representation of salience or hedonic magnitude, or the admixture of distinct representations of positive and negative hedonic values at a spatial scale below the resolution of our images—a question that might be answered using higher resolution imaging techniques.

### Specific Versus Generalized Effects of Attention

Load-related reductions of hedonic responses appear to have been specific to the regions in which they were observed, rather than the result of a global shift in either neural activity or blood flow. This was evidenced by failure of load to modulate the BOLD response to the flickering checkerboard.

### Attentional Modulation of Affective Processing

Our findings are consistent with a growing body of neuroimaging findings indicating that manipulations of attention can modulate neural responses to affective stimuli. Distraction reduces activity in brain regions that respond to pain [47] and to seeing fearful faces [48], and emotional reappraisal (in which people reinterpret the meaning of emotional stimuli in a predetermined way) can regulate ventral striatum and amygdala responses to emotionally-valenced pictures [49]. Other studies have demonstrated a relationship between attention and reward prediction [50,51]. However, to our knowledge, our study is the first to demonstrate attentional modulation of primary appetitive and aversive stimuli.

Importantly, our findings extend previous ones, indicating that manipulations of attention modulate responses concurrently in both cortical and subcortical structures involved in representing the hedonic value of stimuli. This may have significance for understanding the different functions subserved by these structures. For example, one hypothesis is that subcortical structures represent the primary reward value of stimuli, while cortical structures may be important for higher-level forms of valuation (*e*.*g*., those involved in decision making). The question arises as to whether these processes can be dissociated, and thereby independently related to each of these proposed functions.

Affective responding has often been categorized as an “automatic” psychological process [52,53], which implies that it is not influenced by attention. Behaviorally-based assessments such as self-report often rely on this assumption to dismiss the potential influence that the attention required for responding has on the responses themselves. Our observations of attentional modulation of brain areas exhibiting responses to hedonic stimuli raise concerns about this assumption, and the immunity of self-report measures of hedonic events to attentional influence. Similarly, our results also suggest the possibility that subjective hedonic value depends in part on the immediate behavioral goals as mediated through controlled processing. More generally, they demonstrate the widespread influence of attention, suggesting that this extends to experienced utility.

## Supporting Information

S1 Fig Behavioral data. A, Mean Likert rating of how much participants liked the juice and quinine HCl. B, Mean reaction times for the 0-back and 3- back conditions. C, Mean error rate for the 0-back and 3-back conditions. Error bars show s.e.m.
(PDF)

S2 Fig This illustrates how the BOLD level in the 3-back block differed from the 0-back block. The methods and corresponding results were consistent with previous reports on the *n*-back task. A and B: Sagittal slices (x = 6 and x = -38) reveal regions which had larger BOLD in the 3- versus the 0-back condition, including (A) the dorsal anterior cingulate region and (B) the dorsal lateral prefrontal cortex (BA 36 and BA 9), anterior insula, and parietal cortex around BA 40. C (x = 3) shows the opposite (areas in which BOLD is lower in the 3- versus the 0-back conditions). This included large regions in the ventromedial prefrontal cortex and posterior cingulate. We used a significance threshold of *q* = 0.05, 10 contiguous nearest neighbor voxels. Color represents significance as indexed by false discovery rate (see logarithmic color bar).
(PDF)

S3 Fig This axial-oriented montage illustrates where the main of effect of load (0- versus 3-back) was significant. Each red voxel represents that all 16 of the leave-one-participant-out folds were significant (i.e., the intersection of each fold). For illustration purposes, only clusters of at least twenty nearest neighbors are included. (Left = left).
(PDF)

S4 Fig Areas that were significant in the main effect of stimulus (probe, juice, quinine and flickering checkerboard). Each red voxel represents that all 16 of the leave-one-participant-out folds were significant (i.e., the intersection of each fold). For illustration purposes, only clusters of at least ten nearest neighbors are included. (Left = left).
(PDF)

S5 Fig This shows significant regions for the interaction between load and stimulus. Each colored voxel represents that all 16 of the leave-one-participant-out folds were significant (i.e., the intersection of each fold). (Left = left).
(PDF)

S6 Fig Reward processing regions had a load-related reduction of BOLD response to juice and quinine, but visual stimulation was not modulated. A-C shows the effect of the task on brain responses to the solutions, which were found in the regions listed in Table 1. The hemodynamic estimates are provided here for purposes of interpretation. A, the SPM (neurological convention) shows a region in the left striatum (circled) that exhibited significant load-related BOLD reduction. Estimated hemodynamic impulse responses are shown for: quinine for 0- and 3-back blocks in the first column; juice for 0- and 3-back blocks in the second column; and the visual flickering checkerboard for 0- and 3-back blocks in the third column. B, same convention as A but for the right striatum. C, same convention but for a cluster in the dorsal anterior cingulate region. Note that the hemodynamic response estimates may have some statistical dependence on the contrast used to select the regions of interest. These can be used to interpret the contrast, but actual values may be slightly different. The percentage change plotted in this figure is baseline corrected to the first T.R of the relevant condition. Error bars show s.e.m.
(PDF)

S1 Table All the possible trial types, and their probabilities, with respect to the timing of juice, quinine, and flickering checkerboard administration. This shows every way that juice, quinine, and checkerboard can combine within a trial within the constraints that 1) juice and quinine are not delivered on the same trial and 2) no two events will occur at the same time. Probabilities were set such that these three event types occurred on half of all trials. Thus, each occurred with equal frequency (1 out of every 6 trials) at each of the three event time points.
(PDF)

S1 Text Details of analysis on task performance. (PDF)

S2 Text The exit questionnaire that all participants filled out. (PDF)

S3 Text Region of interest analysis on orbitofrontal cortex. (PDF)
