# Supplementary material for: Attentional Modulation of Brain Responses to Primary Appetitive and Aversive Stimuli
Source: PLoS One. 2015 Jul 9;10(7):e0130880. doi: 10.1371/journal.pone.0130880 (PMC4497686; doi:10.1371/journal.pone.0130880)
Supplement: S1 Fig — A, Mean Likert rating of how much participants liked the juice and quinine HCl. B, Mean reaction times for the 0-back and 3- back conditions. C, Mean error rate for the 0-back and 3-back conditions. Error bars show s.e.m. (PDF) [file pone.0130880.s001.pdf]

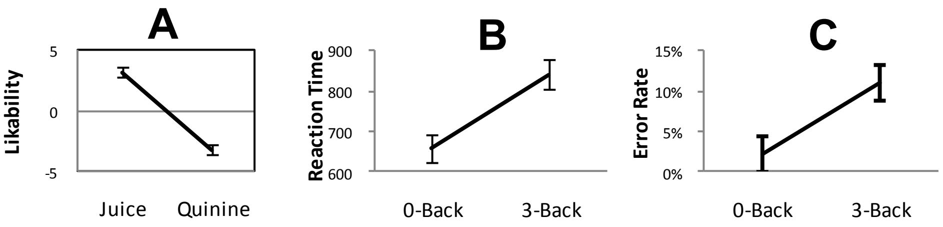

S1 Fig. Behavioral data. A, Mean Likert rating of how much participants liked the juice and quinine HCl. B, Mean reaction times for the 0-back and 3- back conditions. C, Mean error rate for the 0-back and 3-back conditions. Error bars show s.e.m.
