# Supplementary material for: Attentional Modulation of Brain Responses to Primary Appetitive and Aversive Stimuli
Source: PLoS One. 2015 Jul 9;10(7):e0130880. doi: 10.1371/journal.pone.0130880 (PMC4497686; doi:10.1371/journal.pone.0130880)
Supplement: S2 Fig — The methods and corresponding results were consistent with previous reports on the n-back task. A and B: Sagittal slices (x = 6 and x = -38) reveal regions which had larger BOLD in the 3- versus the 0-back condition, including (A) the dorsal anterior cingulate region and (B) the dorsal lateral prefrontal cortex (BA 36 and BA 9), anterior insula, and parietal cortex around BA 40. C (x = 3) shows the opposite (areas in which BOLD is lower in the 3- versus the 0-back conditions). This included large regions in the ventromedial prefrontal cortex and posterior cingulate. We used a significance threshold of q = 0.05, 10 contiguous nearest neighbor voxels. Color represents significance as indexed by false discovery rate (see logarithmic color bar). (PDF) [file pone.0130880.s002.pdf]

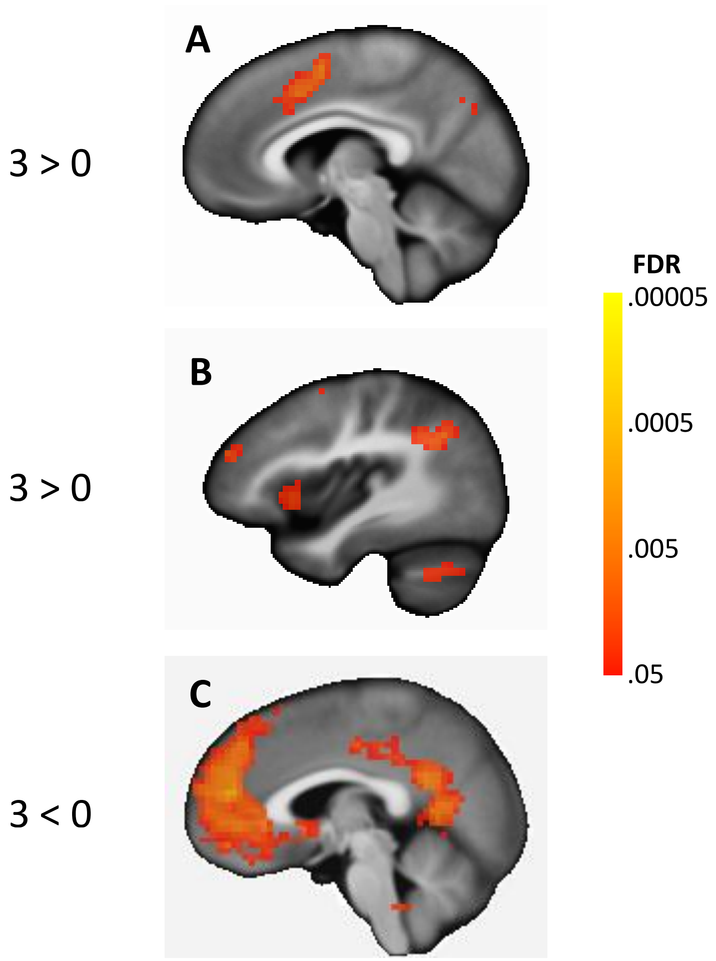

S2 Fig. This illustrates how the BOLD level in the 3-back block differed from the 0-back block. The methods and corresponding results were consistent with previous reports on the *n*-back task. **A** and **B**: Sagittal slices (x = 6 and x=-38) reveal regions which had larger BOLD in the 3- versus the 0-back condition, including (**A**) the dorsal anterior cingulate region and (**B)** the dorsal lateral prefrontal cortex (BA 36 and BA 9), anterior insula, and parietal cortex around BA 40. **C** (x=3) shows the opposite (areas in which BOLD is lower in the 3- versus the 0-back conditions). This included large regions in the ventromedial prefrontal cortex and posterior cingulate. We used a significance threshold of *q* = 0.05, 10 contiguous nearest neighbor voxels. Color represents significance as indexed by false discovery rate (see logarithmic color bar).
