# Supplementary material for: Attentional Modulation of Brain Responses to Primary Appetitive and Aversive Stimuli
Source: PLoS One. 2015 Jul 9;10(7):e0130880. doi: 10.1371/journal.pone.0130880 (PMC4497686; doi:10.1371/journal.pone.0130880)
Supplement: S3 Fig — Each red voxel represents that all 16 of the leave-one-participant-out folds were significant (i.e., the intersection of each fold). For illustration purposes, only clusters of at least twenty nearest neighbors are included. (Left = left). (PDF) [file pone.0130880.s003.pdf]

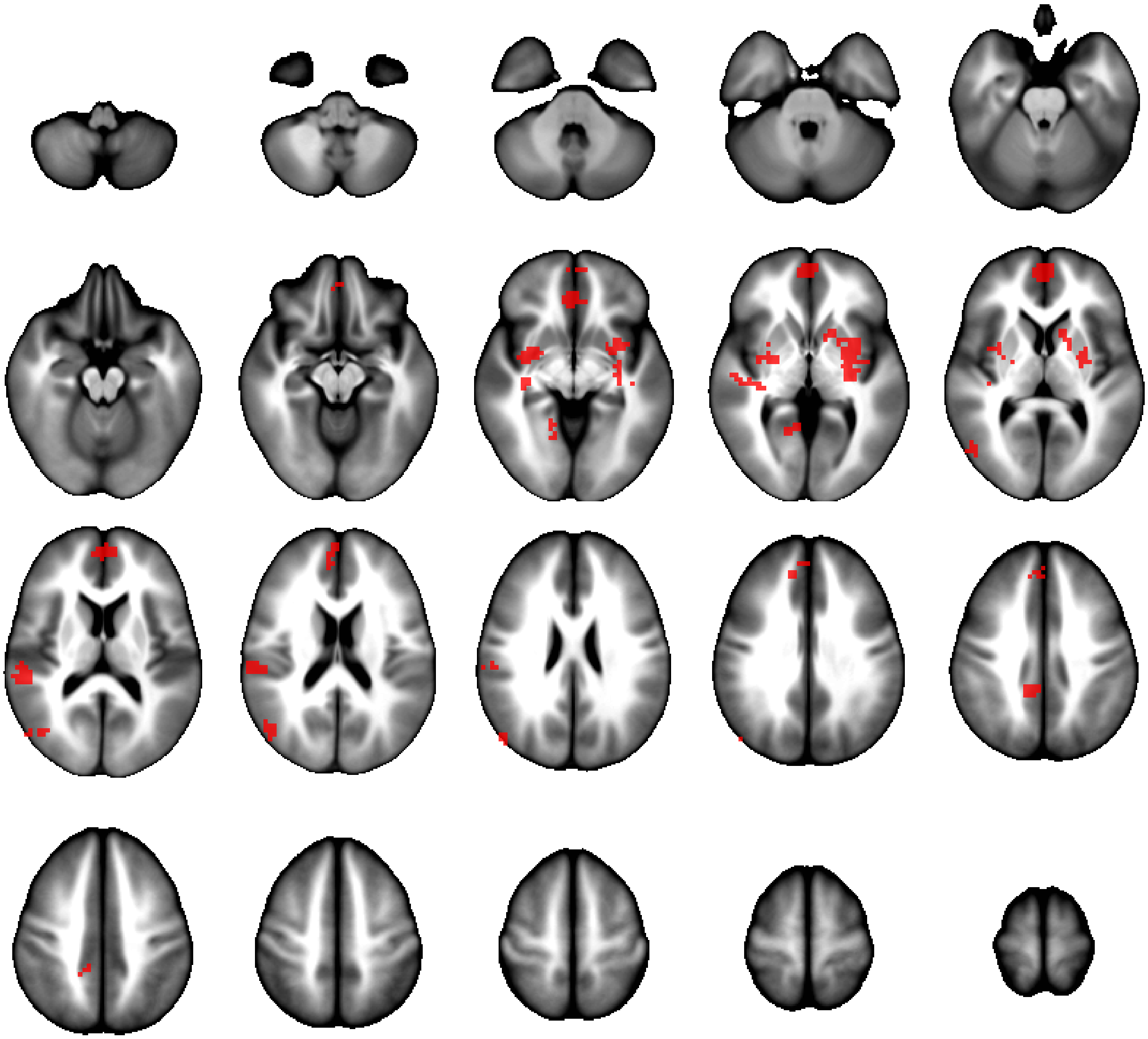

S3 Fig. **This axial-oriented montage illustrates where the main of effect of load (0- versus 3-back) was significant.** Each red voxel represents that all 16 of the leave-one-participant-out folds were significant (i.e., the intersection of each fold). For illustration purposes, only clusters of at least twenty nearest neighbors are included. (Left = left).
