# Supplementary material for: Attentional Modulation of Brain Responses to Primary Appetitive and Aversive Stimuli
Source: PLoS One. 2015 Jul 9;10(7):e0130880. doi: 10.1371/journal.pone.0130880 (PMC4497686; doi:10.1371/journal.pone.0130880)
Supplement: S5 Fig — Each colored voxel represents that all 16 of the leave-one-participant-out folds were significant (i.e., the intersection of each fold). (Left = left). (PDF) [file pone.0130880.s005.pdf]

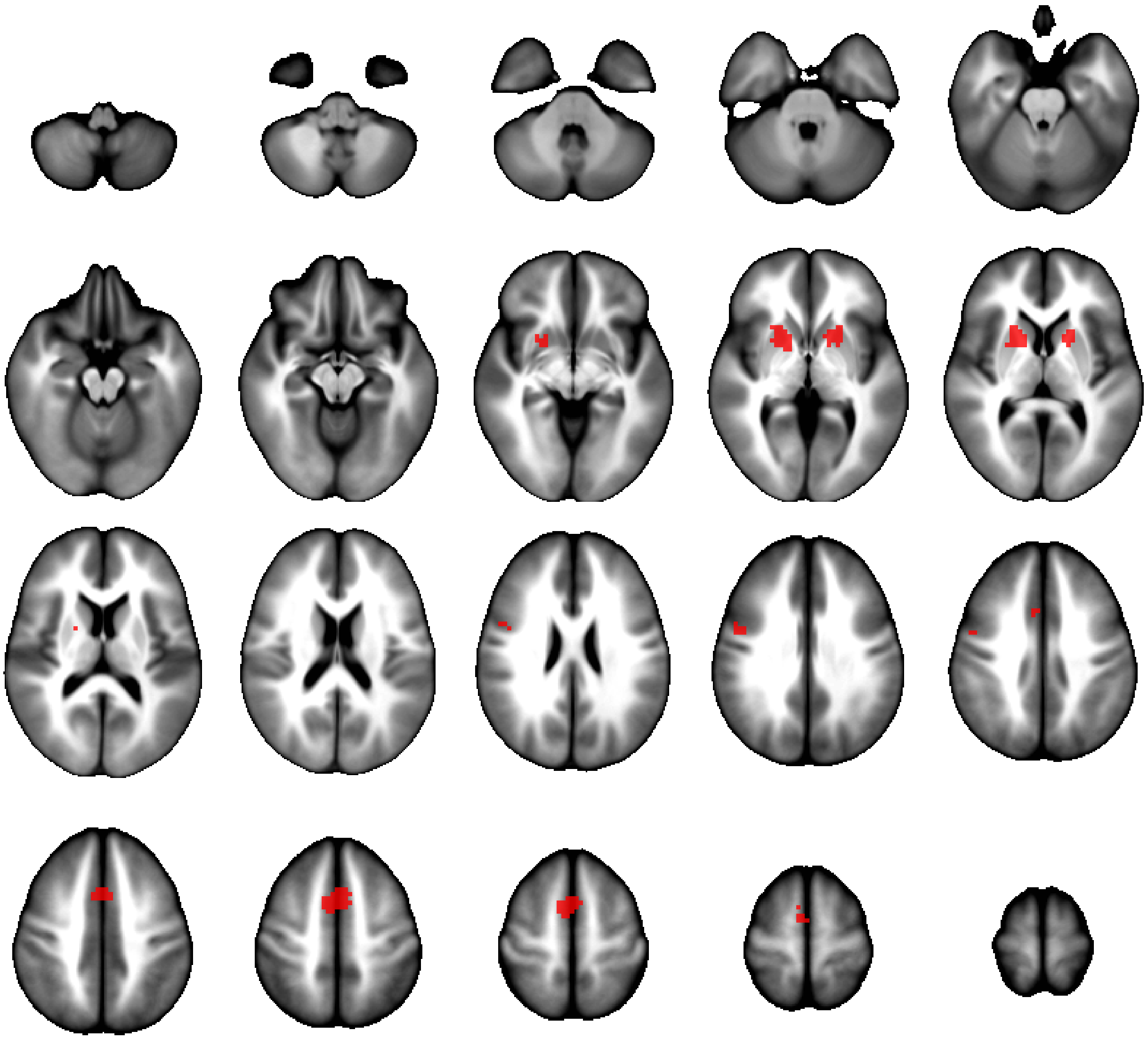

S5 Fig. **This shows significant regions for the interaction between load and stimulus.** Each colored voxel represents that all 16 of the leave-one-participant-out folds were significant (i.e., the intersection of each fold). (Left = left).
