# Supplementary material for: Attentional Modulation of Brain Responses to Primary Appetitive and Aversive Stimuli
Source: PLoS One. 2015 Jul 9;10(7):e0130880. doi: 10.1371/journal.pone.0130880 (PMC4497686; doi:10.1371/journal.pone.0130880)
Supplement: S6 Fig — A-C shows the effect of the task on brain responses to the solutions, which were found in the regions listed in Table 1. The hemodynamic estimates are provided here for purposes of interpretation. A, the SPM (neurological convention) shows a region in the left striatum (circled) that exhibited significant load-related BOLD reduction. Estimated hemodynamic impulse responses are shown for: quinine for 0- and 3-back blocks in the first column; juice for 0- and 3-back blocks in the second column; and the visual flickering checkerboard for 0- and 3-back blocks in the third column. B, same convention as A but for the right striatum. C, same convention but for a cluster in the dorsal anterior cingulate region. Note that the hemodynamic response estimates may have some statistical dependence on the contrast used to select the regions of interest. These can be used to interpret the contrast, but actual values may be slightly different. The percentage change plotted in this figure is baseline corrected to the first T.R of the relevant condition. Error bars show s.e.m. (PDF) [file pone.0130880.s006.pdf]

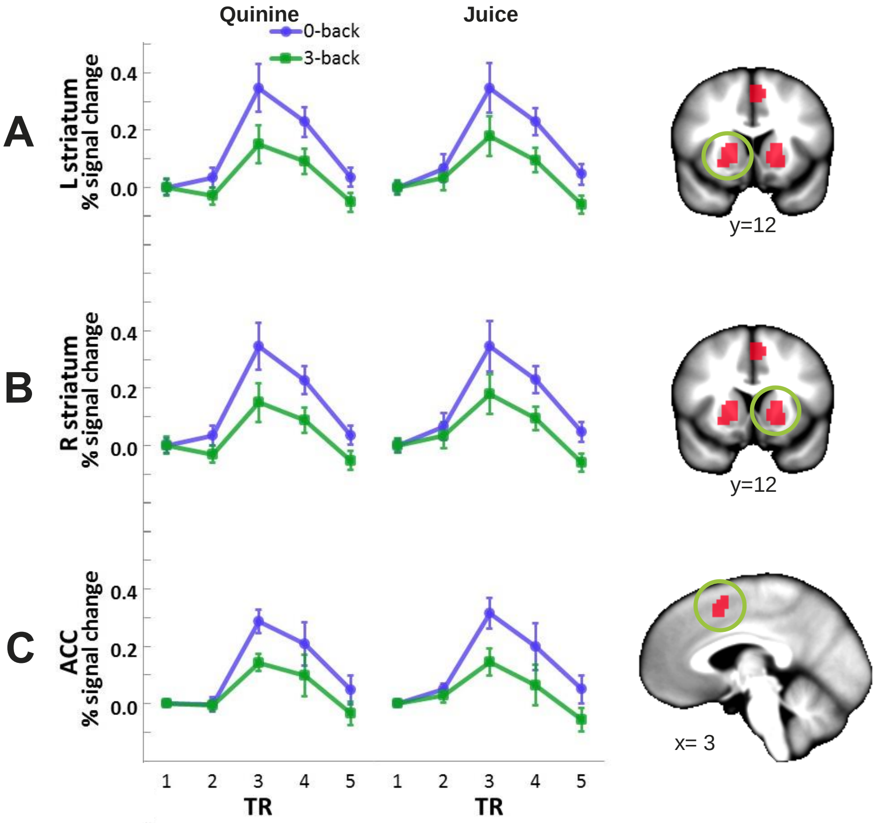

S6 Fig. Reward processing regions had a load-related reduction of BOLD response to juice and quinine, but visual stimulation was not modulated. A-C shows the effect of the task on brain responses to the solutions, which were found in the regions listed in Table 1. The hemodynamic estimates are provided here for purposes of interpretation. **A**, the SPM (neurological convention) shows a region in the left striatum (circled) that exhibited significant load-related BOLD reduction. Estimated hemodynamic impulse responses are shown for: quinine for 0- and 3-back blocks in the first column; juice for 0- and 3-back blocks in the second column; and the visual flickering checkerboard for 0- and 3-back blocks in the third column. **B**, same convention as **A** but for the right striatum. **C**, same convention but for a cluster in the

dorsal anterior cingulate region. Note that the hemodynamic response estimates may have some statistical dependence on the contrast used to select the regions of interest. These can be used to interpret the contrast, but actual values may be slightly different. The percentage change plotted in this figure is baseline corrected to the first T.R of the relevant condition. Error bars show s.e.m.
