# Supplementary material for: Attentional Modulation of Brain Responses to Primary Appetitive and Aversive Stimuli
Source: PLoS One. 2015 Jul 9;10(7):e0130880. doi: 10.1371/journal.pone.0130880 (PMC4497686; doi:10.1371/journal.pone.0130880)
Supplement: S1 Table — This shows every way that juice, quinine, and checkerboard can combine within a trial within the constraints that 1) juice and quinine are not delivered on the same trial and 2) no two events will occur at the same time. Probabilities were set such that these three event types occurred on half of all trials. Thus, each occurred with equal frequency (1 out of every 6 trials) at each of the three event time points. (PDF) [file pone.0130880.s007.pdf]

Entry	Prob.	6 seconds	Trial Time<br>8 seconds	10 seconds
1	0.083	Juice		
2	0.083		Juice	
3	0.083			Juice
4	0.042	Juice		Checkerboard
5	0.042		Juice	Checkerboard
6	0.042	Juice	Checkerboard	
7	0.042		Checkerboard	Juice
8	0.042	Checkerboard	Juice	
9	0.042	Checkerboard		Juice
10	0.083	Quinine		
11	0.083		Quinine	
12	0.083			Quinine
13	0.042	Quinine		Checkerboard
14	0.042		Quinine	Checkerboard
15	0.042	Quinine	Checkerboard	
16	0.042		Checkerboard	Quinine
17	0.042	Checkerboard	Quinine	
18	0.042	Checkerboard		Quinine
	∑=1.00			

S1 Table. All the possible trial types, and their probabilities, with respect to the timing of juice, quinine, and flickering checkerboard administration. This shows every way that juice, quinine, and checkerboard can combine within a trial within the constraints that 1) juice and quinine are not delivered on the same trial and 2) no two events will occur at the same time. Probabilities were set such that these three event types occurred on half of all trials. Thus, each occurred with equal frequency (1 out of every 6 trials) at each of the three event time points.
