# Supplementary material for: Attentional Modulation of Brain Responses to Primary Appetitive and Aversive Stimuli
Source: PLoS One. 2015 Jul 9;10(7):e0130880. doi: 10.1371/journal.pone.0130880 (PMC4497686; doi:10.1371/journal.pone.0130880)
Supplement: S1 Text — (PDF) [file pone.0130880.s008.pdf]

S1 Text. **Details of analysis on task performance**.

For the first eleven subjects, responses were recorded only for the first 1.25 s post stimulus onset. However, we suspected that subjects were occasionally taking longer than 1.25 s to respond in the high-load task. To assure that subjects were maintaining high response accuracy, we extended the response recording period to 3.0 s for the remaining 6 subjects. This modification had no impact on subjects' performance. Subjects were slower on the 3-back task. Subjects who had the short response window: $F(1,1046) = 31.46$, $p=1.92e-04$; long response window: $F(1,688)=30.51$, $p=1.73e-02$; and all subjects combined: $F(1,1734) = 15.21$, $p=1.00e-04$. Similarly, subjects made more error in the 3-back task. Subjects who had the short response window: $F(1,1298) = 34.87$, 1.50e-04; long response window: $F(1,708) = 4.41$, $p=3.60e-02$; and all subjects combined: $F(1,2039)=28.45$, $p=7.00e-05$.
