# Supplementary material for: Attentional Modulation of Brain Responses to Primary Appetitive and Aversive Stimuli
Source: PLoS One. 2015 Jul 9;10(7):e0130880. doi: 10.1371/journal.pone.0130880 (PMC4497686; doi:10.1371/journal.pone.0130880)
Supplement: S2 Text — (PDF) [file pone.0130880.s009.pdf]

Exit Questionnaire Subject Number:_________

Please rate your response to the following questions by placing a checkmark in the appropriate box.

| | Not at all | | | | | | | | | | Very Much |
|---|---|---|---|---|---|---|---|---|---|---|---|
| How thirsty were you when you arrived? | -5 | -4 | -3 | -2 | -1 | 0 | +1 | +2 | +3 | +4 | +5 |
| How thirsty are you now? | -5 | -4 | -3 | -2 | -1 | 0 | +1 | +2 | +3 | +4 | +5 |
| How much did you enjoy the juice? | -5 | -4 | -3 | -2 | -1 | 0 | +1 | +2 | +3 | +4 | +5 |
| How much did you enjoy the tonic water? | -5 | -4 | -3 | -2 | -1 | 0 | +1 | +2 | +3 | +4 | +5 |

**Text S2. The exit questionnaire that all participants filled out.**
