# Supplementary material for: Attentional Modulation of Brain Responses to Primary Appetitive and Aversive Stimuli
Source: PLoS One. 2015 Jul 9;10(7):e0130880. doi: 10.1371/journal.pone.0130880 (PMC4497686; doi:10.1371/journal.pone.0130880)
Supplement: S3 Text — (PDF) [file pone.0130880.s010.pdf]

S3 Text. Region of interest analysis on orbitofrontal cortex

There is considerable EPI signal dropout in orbitofrontal cortex due to differences in magnetic susceptibility at the air-tissue boundary of the sinuses. This adds considerable region-specific noise to gradient-echo echo-planer images and thus weakens statistical analyses based on them. Such signal dropout could have masked a value signal in orbitofrontal cortex.

We employed a Region of Interest (ROI) analysis to evaluate whether the orbitofrontal cortex represented value and, if so, the extent to which this was modulated by load. To define the ROI we used the region labelled “Superior Orbital Gyrus” from the Eickoff-Zilles “macro labels” atlas that comes bundled with AFNI. The ROI overlaps the orbital gyri with some extension into neighboring tissue, but largely avoids the rectal gyrus.

We excluded subjects who had poor signal in the ROI. We defined poor ROI signal to be when fewer than 60% of the voxels in the ROI fall within the volume of “good” signal according the AFNI program 3dAutomask. 3dAutomask by default defines “good” as anything above the median of all (non-zero) values. However, some small residual distortions remain at the edges of the volume.

Using this criterion we excluded four subjects. With these subjects excluded, there was a trend-level effect of valence in the left orbitofrontal ROI ($p=0.097$). Juice produced stronger BOLD responses than quinine. As for other regions we report on, the BOLD response to the combination of juice and quinine was significantly reduced by load ($p = 0.044$). Both juice ($p=0.092$) and quinine ($p=0.076$) independently showed a trend-level reduction in response due to load.
